# De Novo Genes: Current Status and Future Goals

**DOI:** 10.1093/gbe/evaf230

**Published:** 2025-11-28

**Authors:** Claudio Casola, Victor Luria, Nikolaos Vakirlis, Li Zhao

**Affiliations:** Department of Ecology and Conservation Biology, Texas A&M University, College Station, TX 77845, USA; Interdisciplinary Doctoral Degree Program in Ecology and Evolutionary Biology, Texas A&M University, College Station, TX 77845, USA; Interdisciplinary Doctoral Degree Program in Genetics and Genomics, Texas A&M University, College Station, TX 77845, USA; Department of Neuroscience, Yale University School of Medicine, New Haven, CT, USA; Division of Genetics and Genomics, Boston Children’s Hospital, Harvard Medical School, Boston, MA 02115, USA; Program in Medical and Population Genetics, Broad Institute of Harvard and MIT, Cambridge, MA 02142, USA; Hellenic Pasteur Institute, Athens 11521, Greece; Laboratory of Evolutionary Genetics and Genomics, The Rockefeller University, New York, NY 10065, USA

**Keywords:** comparative genomics, gene evolution, microproteins, ribosome profiling, random peptides, genome evolution

## Abstract

The recent Society for Molecular Biology and Evolution Satellite Meeting on De Novo Gene Birth, hosted at Texas A&M University on November 6 to 9, 2023, represented the first-ever opportunity for scientists studying the evolution and biology of de novo genes to gather through a dedicated meeting and discuss about groundbreaking discoveries in this emerging and exciting field of gene evolution. In this perspective, we discuss recent advances and major open questions in de novo gene emergence and evolution that were presented at the SMBE satellite meeting, as well as some of the key recent findings published before or since the conference. These key themes include de novo gene identification, function, and evolution, what we are learning about de novo genes from experimental analyses of random peptides, de novo gene birth and microproteins, and the role of de novo genes in human disease.

Significance De novo gene birth—the emergence of genes from previously noncoding DNA—has moved from a once-dismissed concept to a recognized driver of evolutionary novelty, yet many aspects of this process remain poorly understood. Drawing on discussions and findings from the first dedicated international meeting on de novo gene birth, we highlight recent advances in detecting these genes, characterizing their molecular and functional properties, and uncovering their roles in adaptation, disease, and the origin of microproteins. By synthesizing these developments and outlining pressing open questions, this perspective provides a roadmap for integrating genomic, functional, and structural approaches to fully reveal how de novo genes shape genome evolution and organismal biology.

## What are De Novo Genes?

The origin of new genes—operationally defined here as protein-coding genes—is a core question in molecular evolution. Gene duplication and horizontal gene transfer, the latter being a prominent process especially among bacteria and archaea, have long been recognized as primary mechanisms giving rise to novel genes (Long et al. 2003). However, the abundance of non-genic DNA, especially in eukaryotic genomes, raises the possibility that new genes could originate from these regions. De novo gene birth is a recently described evolutionary process that answers both these questions. In general terms, de novo protein-coding genes evolve from DNA that is ancestrally non-coding. This may be extended to include novel protein-coding sequences that originate from alternative reading frames of existing coding regions, a phenomenon known as overprinting that is well-studied in viruses (Grassé 1977; Sabath et al. 2012) and is known to also occur in eukaryotes (Vasu et al. 2022).

De novo genes were first posited by Stephens (Stephens 1951; Tautz 2014), but the idea was generally dismissed, notably by François Jacob, as highly improbable (Jacob 1977). In the 1990s, several studies on orphan genes and overlapping genes suggested the possibility of de novo gene birth (Keese and Gibbs 1992; Dujon 1996; Domazet-Loso and Tautz 2003). Furthermore, genes encoding antifreeze glycoproteins in the Antarctic notothenioid fish were reported to be partially formed by de novo coding regions originating from microsatellites (Chen et al. 1997), a mechanism of de novo gene birth that led to the convergent evolution of antifreeze glycoproteins in the Arctic gadid family (Zhuang et al. 2019). However, the first unquestionable discovery of the de novo genes dates back to the early 2000s, when critical comparative genomic resources become increasingly available in model taxonomic groups, such as *Drosophila* (Begun et al. 2006; Levine et al. 2006), great apes (Knowles and McLysaght 2009; Li et al. 2010a), mouse (Heinen et al. 2009; Linnenbrink et al. 2024), and budding yeasts (Cai et al. 2008; Li et al. 2010b). Typically, a gene is considered to have evolved de novo when it shares no sequence similarity at statistically meaningful thresholds (typically, e-value < 0.001) with genes from outgroup species and when it can be inferred that ancestrally its locus lacked protein-coding capacity, either that it was expressed but contained no sufficiently long open reading frame (ORF) existed, or that it was not expressed. Changes in promoter regions may also facilitate the transcription of the new locus, regardless of its coding potential.

Interest in de novo gene birth has significantly grown in the past two decades. Major findings in this area have been described in depth in several recent reviews and will not be covered here (McLysaght and Hurst 2016; Van Oss and Carvunis 2019; Bornberg-Bauer et al. 2021; Broeils et al. 2023; Zhao et al. 2024). Rather, in this perspective, we aim at pointing out the major areas of discovery and the fundamental questions on de novo gene birth still lingering, which makes this field one of the most exciting areas in molecular evolution.

## Identifying De Novo Genes

Surveys of de novo genes can rely on annotated gene sets or take advantage of novel transcriptomic or ribosome profiling data to identify recently evolved loci encoding unannotated transcripts and proteins. Either approach identifies de novo genes within a larger set of taxonomically restricted genes (TRGs) that share no similarity at the sequence level beyond a predefined taxonomic range (eg a species, genus, or family of focus). TRGs are usually identified using a phylostratigraphic approach consisting of sequence homology analyses along species phylogenies and the resulting patterns of gene presence/absence (Domazet-Loso et al. 2007). While this approach is relatively straightforward and offers a suitable list of candidate TRGs, it does not provide sufficient information to identify the specific mechanism of origin of lineage-specific genes (Pereira et al. 2025), although it largely rules out duplication since recently duplicated genes appear as ancient as the gene from which they were copied. The best evidence that a given TRG emerged de novo comes from identification and careful analysis of its orthologous loci in closely related outgroup genomes that can be reliably aligned to the genome of the focal species, allowing one to infer with relative confidence that the locus was ancestrally noncoding. Many examples of de novo genes provided such evidence in the form of alignments of nongenic orthologous loci to the TRG in question, showing cases of enabling substitutions, which derive from point mutations or small indels that, based on parsimony, could be shown to have caused the formation of the ORF of the TRG of interest. For instance, high-confidence de novo genes were discovered in the rice *O. sativa* subspecies *japonica* integrating genomic data from 13 *Oryza* species, identifying indels as major mutations responsible for generating longer ORFs (Zhang et al. 2019).

In recent years, some studies have improved upon this manual, parsimony-based approach, by performing ancestral sequence reconstruction (ASR) using as input a multiple sequence alignment of a TRG of interest to its orthologous sequences in neighboring species, in order to infer ancestral positions or entire ancestral sequences based on evolutionary models (Vakirlis et al. 2020, 2022; Lange et al. 2021; Papadopoulos et al. 2021; Sandmann et al. 2023; Peng and Zhao 2024). Once an ancestral sequence has been reconstructed, the presence or absence of an ORF of interest can be assessed and one can then conclude about whether or not it appears to have emerged de novo. The potential of ASR for the study of de novo gene birth is high, both for robustly identifying de novo genes and for tracing the evolutionary path a noncoding sequence might take toward protein-coding capacity (Vakirlis et al. 2024). Yet various potential pitfalls exist, because we do not yet have enough “ground truth” cases of de novo genes to allow the optimal tuning of ASR-based methodologies. One concern is pinpointing the exact evolutionary timing of the birth of a particular extant ORF, given a series of reconstructed ancestral ORFs of variable lengths, more or less close to that of the extant one. Given that the details of the process of de novo gene birth remain unclear, it is challenging to justify why an ancestral ORF inferred through ASR making up—for example—50% of an extant one's length should not count as “existing” but one at 70% should. This issue is compelled by different views on what constitutes a gene, and by the lack of comparative functional studies on syntenic ORFs of various lengths. Furthermore, more studies on sequence alignment uncertainties and DNA evolutionary models are needed to improve our inferences of de novo gene evolution. A recently originated de novo gene may also easily become lost again in some lineages, adding difficulty to the task of accurately reconstructing its origin. Finally, ASR is limited by the conservation of microsynteny, which for most genes in vertebrates lasts for several hundreds of millions of years while in rapidly evolving taxa, including some insects and many angiosperms, it can be quickly erased.

Identifying a gene as de novo emerged means convincingly excluding alternative evolutionary scenarios. One such alternative is the pseudogenization/loss of de novo genes in outgroup lineages. This can lead to a gene appearing as TRG (as there might be no detectable similarity to orthologues in other genomes) and meeting the above-mentioned criteria to qualify as de novo (orthologous sequence present and without an ORF, in multiple species). To account for such a scenario, it is important to perform similarity searches against six-frame translations of entire genomes, both the focal species’ own and outgroup ones. Nevertheless, these approaches have the caveat that the number of ORFs in six frames is very large, and the vast majority of them have no evidence of transcription or translation.

Computational pipelines that integrate these criteria for large-scale detection of de novo genes have recently emerged, facilitated by extensive comparative genomic resources, faster homology search tools (Buchfink et al. 2021), and strict control of homology biases. For example, the GenEra tool provides estimates of new gene age and has been critical to assess genome innovation in specific lineages (Barrera-Redondo et al. 2023). GenEra corrects for homology loss due to rapid evolution of orthologous sequences, or homology detection failure (Weisman et al. 2020), improving the accuracy of gene age estimates. Specifically, this method examines the long-term change in sequence similarity, while otherwise being limited by loss of synteny for the purposes of true homology detection. While GenEra and similar tools are not sufficient to detect de novo genes, they provide robust catalogs of lineage-specific genes that can be further examined to search for substitutions associated with de novo gene birth. The suite DENSE was specifically developed to detect de novo genes (Roginski et al. 2024). These novel tools and more refined strategies will soon lead to comprehensive catalogs of de novo genes across multiple taxa. Integrated with other genomic information, these datasets will allow scientists to explore in an evolutionary framework fundamental but poorly understood features of de novo genes, such as rates of formation and loss, functional impact, and sequence evolution.

## Origin and Evolution of De Novo Genes and Proteins

How and in what order the various gene-like characteristics originate and become fixed in natural populations is an outstanding question regarding de novo genes. Analyses in several organisms have shown that transcription of non-coding DNA, essentially lncRNAs, often precede the formation of a longer ORF (Wu et al. 2011; Carvunis et al. 2012; Zhao et al. 2014; Ruiz-Orera et al. 2015), although it remains unclear whether this is a lineage-biased pattern or if it is related to population structure or regulatory architecture of the species. In *S. cerevisiae*, the property of de novo genes to encode proteins with transmembrane (TM) domains is inherited from their non-genic precursors in what can be described as a TM-first case of emergence (Vakirlis et al. 2020). This is due to polyA/T tracts that are abundant and enriched within intergenic regions (Vakirlis and Fuqua 2025) and is likely true in many other species of yeast as well (Tassios et al. 2023). The case of *S. cerevisiae* serves as an example of how biases within the noncoding genome can affect the functional properties of de novo genes, although this specific bias has so far only been found in this species and it is unclear if similar ones exist within other genomes. Population genomic data have increasingly been analyzed to address these questions. For example, the analysis of inbred lines of *D. melanogaster* has revealed a rapid turnover of segregating de novo expressed ORFs (neORFs), or de novo gene precursors, largely due to variation in transcription of neORFs between lines (Grandchamp et al. 2023). High levels of de novo gene expression polymorphism have been also reported in natural North American and Zambian populations of *D. melanogaster*, with a substantial proportion of these population-specific de novo genes encoding predicted secreting proteins (Cridland et al. 2025). Simulation studies implementing a range of mutation rates and mutation effects, several of which were presented at the meeting, have also reinforced the view that de novo genes are more likely to evolve in the proximity of other genes by exploiting existing regulatory regions (Lee et al. 2024, 2025), while indicating that high rates of de novo gene loss should be expected (Mani and Tlusty 2023).

A further primary goal of de novo gene studies is to determine how they evolve through time. A widespread view is that increasingly older de novo genes—as a category, not as individual genes—should become structurally and functionally more similar to typical genes, for example, expanding their coding sequence via the acquisitions of new exons, increasing their expression breadth, and encoding proteins that are less disordered and mirror more closely the amino acid composition of other the canonical proteome. These and other features have been analyzed in recent works across eukaryotic model species (An et al. 2023; Chen et al. 2024; Papadopoulos et al. 2024; Peng and Zhao 2024). However, a comprehensive understanding of de novo gene evolution will require broadening the taxonomic range of investigated genomes and integrating more sophisticated models of sequence evolution into analyses of de novo gene and protein features.

## De Novo Gene Function

Understanding the function of de novo genes is an essential question in the field, not only because it sheds light on their physiological roles in biology and human disease, but also because it provides important insights into evolution. Several de novo genes have been experimentally characterized and shown to play important biological functions, including but not limited to cases described in *Drosophila* (Lange et al. 2021; Rivard et al. 2021), angiosperms (Li et al. 2009; Chen et al. 2023; Jin et al. 2025), mouse (Heinen et al. 2009; Xie et al. 2019; Linnenbrink et al. 2024), and *Saccharomyces* (Li et al. 2010b, 2014; Vakirlis et al. 2020). De novo genes with a plausible role in human neurogenesis have also been reported (An et al. 2023), although follow-up studies have debated their exact level of taxonomic restriction and reassigned some from species-specific (humans) to class-specific (mammals) (Leushkin and Kaessmann 2024; Xiao et al. 2024). These cases exemplify some of the major challenges associated with the discovery, phylogenetic placement, and functional validation of de novo genes. Most de novo genes described by An et al. (2023) are formed by short open reading frames often found within or next to other coding region that appear to be well conserved outside of hominoids. The limited evidence of translation, occurrence within other genes, and lack of data on enabler mutations led to the dismissal of many cases as non-coding sequences (Leushkin and Kaessmann 2024). However, more comprehensive proteomic and translatomic data, together with the identification of potential enabler mutations, indicate that many of these ORFs might represent de novo genes (Xiao et al. 2024). This debate illuminates the importance of supporting proposed de novo genes with extensive comparative and functional genomic data, particularly given the ability of Ribo-seq analyses to provide large catalogs of novel short coding regions within existing loci.

Sequence analyses have shown selective constraints of de novo gene coding regions in some cases, which is suggestive of a functional impact (Zhao et al. 2014; Gubala et al. 2017; Vakirlis et al. 2018; Heames et al. 2020; Cridland et al. 2022; Montanes et al. 2023; Wacholder et al. 2023). However, sequence conservation inferences have limited power in the typically short de novo genes. Furthermore, lack of sequence conservation does not necessarily mean lack of function. For instance, systems biology approaches in budding yeast revealed thousands of species-specific potential de novo genes might be consistently translated, with some of them exhibiting a phenotypic impact when knocked-out, though they mostly show no signatures of sequence evolutionary constraints (Wacholder et al. 2023). It remains unclear if this “transient translatome” represents a common feature of young de novo genes, hinting at a limited and labile evolutionary impact of such genes. Indeed, de novo genes experience high turnover rates and rapid loss (Palmieri et al. 2014; Zhao et al. 2014; Neme and Tautz 2016). Nevertheless, studies in *Drosophila* (Chen et al. 2010; Peng and Zhao 2024), budding yeast (Carvunis et al. 2012; Blevins et al. 2021), humans (Chen et al. 2015; Guerzoni and McLysaght 2016), and rice (Zhang et al. 2019) have shown that many de novo genes persist for long evolutionary times, and knockdown experimental data from *D. melanogaster* point to young genes, including de novo genes, quickly gaining essentiality (Chen et al. 2010; Xia et al. 2021). Altogether, these findings suggest that some de novo genes can persist over long evolutionary periods and become critical to organismal’ fitness.

Investigating the regulation of de novo gene expression is also essential to determine their functional role as well as their evolution from DNA that is originally “silent.” In various species, these genes are often biased toward specific tissues such as the testis (Ruiz-Orera et al. 2015; Cridland et al. 2022), the immune system (Peng and Zhao 2024), and the brain (Li et al. 2010a, 2010b; Wu et al. 2011; Zhang et al. 2011). For example, single-cell sequencing data to demonstrate that de novo gene expression can be highly dynamic at the cellular level, suggesting that the regulatory machinery of novel genes might be complex (Witt et al. 2019). Understanding how cis- and trans-regulatory elements coordinate to establish these novel expression patterns is thus crucial. A recent study using deep learning techniques revealed that novel open chromatin, which is frequently associated with novel genes, often originates from regions that contain some regulatory properties (Khodursky et al. 2023). This suggests that chromatin state transitions are constrained at the chromosomal level, potentially influencing the chromosomal localization of de novo genes. Additionally, young genes, including de novo genes, show different 3D genomic positioning compared to established genes that affect their tissue-specific expression (Fleck et al. 2024). Furthermore, proteins encoded by de novo genes in vertebrates are also expected to be expressed in the early stages of their evolution in tissues mediating the recognition of self and non-self antigens, to avoid autoimmune reactions (Bekpen et al. 2018). Accordingly, intergenic transcripts, which experience rapid turnover and can evolve into de novo genes, are often expressed in the thymus and spleen in mice species (Bekpen et al. 2018).

A recent work based on massively integrated coexpression analysis in budding yeast found that noncanonical ORFs often co-express with canonical ORFs, a pattern also observed in de novo ORFs, especially with respect to their upstream neighbor genes (Rich et al. 2024). These coexpression patterns imply that half of de novo ORFs might be functionally associated with conserved genes, contributing to specific biological functions. Similarly, in *Drosophila*, many de novo long non-coding RNAs (lncRNAs) are located near conserved genes (Lee et al. 2024). These lncRNAs may be fixed and maintained through their linkage with conserved genes. This proximity suggests a potential functional association, further highlighting the importance of considering chromosomal context in the study of novel gene regulation (Lee et al. 2024). Importantly, these works suggest that the expression and functional relationship between novel genes (whether protein coding or lncRNAs) and conserved genes are complex and beyond simply sharing regulatory elements (Gotea et al. 2013).

Further work will be essential to broadly elucidate how de novo genes integrate into existing cellular networks without disrupting them and to determine if de novo genes exhibit biases in the types of cellular or organismal functions they are involved across multiple species. The functional integration of de novo genes might be driven by genomic conflicts (McLysaght and Hurst 2016), as in the case of several gene duplicates (Castellanos et al. 2024); however, this possibility has yet to be demonstrated.

## Exploring De Novo Protein Evolution and Function Using Random Peptides

Genome sequences, including intergenic sequences, are not random. They represent only a small fraction of all possible sequence space. Consequently, potential novel ORFs from real genomes are also not random. The full significance of the sequence space of non-genic sequences is not entirely understood, making the potential of genome-sequence-derived novel ORFs an open question. In this context, studying random ORFs can be informative, as it helps assess the intrinsic functional potential of arbitrary sequences and offers insights into how completely new ORFs might evolve novel functions

Some early work showed that synthetic random ORFs might have a fitness effect both in *E. coli* (Neme et al. 2017) and in human cell lines (Aldrovandi et al. 2024), with most effects being detrimental. Other random peptides have demonstrated functions such as immune responses (Knopp et al. 2019). In another recent study, a synthetic novel ORF was able to rescue a deleterious effect caused by a toxin, suggesting that synthetic ORFs can be integrated into protein homeostasis pathways under strong selective pressure (in this case, artificial selective pressure caused by the toxin) (Frumkin and Laub 2023). Furthermore, it has been shown that the formation of stable globular protein folds can rapidly evolve through a few amino acid replacements of random sequences and that the same fold may be mutationally accessed from multiple initial sequences (Sahakyan et al. 2025).

There are still many intriguing questions to be answered in this field. For example, are biologically generated novel ORFs and synthetic novel ORFs fundamentally similar in their functional potential and properties? In terms of percentage, how comparable are they in producing biologically meaningful products? Since most synthetic ORFs are driven by specific promoters and thus this type of experiment does not answer regulatory origin of novel genes, what roles do novel promoters play? These are all fascinating questions that warrant further exploration in the future.

Comparing the sequences and structural properties of peptides encoded by random ORFs and by de novo genes can also inform on the evolutionary dynamics of the latter. A recent work found that de novo proteins may have similar distributions of biophysical properties to random sequences of a given length and amino acid composition (Heames et al. 2023). However, upon expression in vitro, de novo proteins exhibit moderately higher solubility, which is further enhanced by the DnaK chaperone system. These findings suggest that de novo proteins are better suited, and possibly selected for, features that facilitate their recruitment within cellular networks compared to their random counterparts.

## De Novo Genes as a Major Source of Microproteins

Our understanding of the coding potential of genomes has been revolutionized by the development and refinement of techniques for the high-throughput sequencing of proteins (Hunt et al. 1986) and of RNAs bound to ribosomes (Ingolia et al. 2009). The latter methodology, known as ribosome profiling or Ribo-seq, has been instrumental to the discovery of numerous potentially protein-coding transcripts encoded by nonannotated, or noncanonical, loci. The majority of noncanonical loci products are known as microproteins, to emphasize their shorter length compared to most proteins encoded by annotated “canonical” genes. An important emerging trend is that a significant proportion of microproteins appears evolutionary young and thus could have originated de novo. This comes mostly from investigations in the human lineage (Vakirlis et al. 2022; Sandmann et al. 2023; Ruiz-Orera et al. 2024) but evidence in other species such as *Drosophila* points to it being true more generally (Patraquim et al. 2022). Note that clearly not all microproteins are de novo or even evolutionarily novel, and indeed some are as ancient as canonical proteins. By definition, however, de novo proteins likely start out small and therefore might be microproteins (Patraquim et al. 2022). Thus, the two fields naturally intersect.

Functional noncanonical proteins have been described in multiple taxa (Hanada et al. 2013; Magny et al. 2013; Anderson et al. 2015; Graeff et al. 2016; D'Lima et al. 2017; Azam et al. 2025); however, the biological impact of the noncanonical, or “dark,” proteome remains poorly understood. There is thus an increased interest in annotating and characterizing noncanonical proteins, which is reflected by the numerous contributions on the topic at the satellite meeting on de novo gene birth. One result of this effort is the conclusion that the integration of methods including proteomic, Ribo-seq, transcriptomic and evolutionary conservation analyses produce more reliable annotation of microproteins (Khitun and Slavoff 2019; Ruiz-Orera and Albà 2019; Chothani et al. 2022; Mudge et al. 2022; Schlesinger and Elsässer 2022; Wacholder et al. 2023; Ruiz-Orera et al. 2024; Yang et al. 2024). Microproteins functionality is also supported by expression across multiple tissues (Martinez et al. 2020) and by phenotypic effects upon knockout experiments (Chen et al. 2020; Pai et al. 2025) for many of them. However, microproteins could also be less stable than canonical proteins (Kesner et al. 2023; Casola et al. 2024; Yang et al. 2024). It remains unclear if de novo and non-de novo microproteins exhibit any functional or structural difference that can explain variation in their biological impact. Further noncanonical de novo ORF datasets will be essential to explore this aspect.

Microprotein discovery is a particularly exciting area in eukaryotes due to the enormous genome size of many eukaryotic species. Nevertheless, some of the most poignant discoveries in this area have been made in bacteria. For instance, several functional microproteins have been identified in *E. coli* (Hemm et al. 2008; Aoyama et al. 2022). Additionally, large-scale analyses have shown that among 5,668 genomes from the Enterobacteriaceae family, there are nearly 70 thousand clusters of intergenic smallORFs (15 to 70 codons long) with evidence of purifying selection (Fesenko et al. 2025). Alternative reading frames can represent a primary source of novel small proteins in the compact genomes of bacteria and archaea (Ardern 2023).

## De Novo Genes and Disease

One way to glimpse the function of de novo genes and more broadly of orphan genes is by assessing if mutations or amplifications in such genes are involved in human disease if changed in individuals by mutation or amplification. *NCYM* is a human-specific small proto-oncogene for human neuroblastoma and is translated from an antisense long noncoding RNA of the *MYCN* proto-oncogene (Suenaga et al. 2014). The NCYM protein has 109 amino acids (AA) and its structure is partially folded with dynamic helixes (Mouhand et al. 2024). NCYM stabilizes MYC by inhibiting GSK3β and promotes tumor formation upon amplification and overexpression (Suenaga et al. 2020). More generally, several de novo genes appear to play a role across different types of cancer. For instance, a recent work described 37 de novo genes found only in humans or hominoids that function as oncogenes and could in some cases be developed as antigens for immunotherapy (Xiao et al. 2025). Several other human-specific de novo genes with a role in a range of diseases have been described in a recent review (Broeils et al. 2023). For example, *C20orf203* is a de novo gene highly expressed in Alzheimer's brain samples that might play a role in this disease (Li et al. 2010a, 2010b). Other young genes whose de novo origin remain uncertain have been found in association with human disease. For example, *SPANXA2* is a primate specific X-linked gene encoding a 97 AA protein that binds nuclear pore components associated with poor prognosis in breast cancer and in head and neck squamous cell carcinoma (Li et al. 2020). *C11orf95*, is a primate-specific 47 AA proto-oncogene prone to fusion with the RELA oncogene and subsequently drives ependymal tumors (Zheng et al. 2021). *Myoregulin* (MLN) encodes a Mammal-specific 46 AA protein expressed in muscle that impedes uptake of calcium into the sarcoplasmic reticulum, regulates muscle physiology (Anderson et al. 2015) and appears dysregulated in a dog model of Duchenne muscle dystrophy (Morales et al. 2024). These examples highlight the biological impact of recently evolved protein-coding sequences in fundamental cellular processes and point to the importance of characterizing the phenotypic effects of de novo genes in our species.

## Conclusions

The recent SMBE satellite meeting on de novo gene birth has brought together nearly one hundred researchers from eleven countries, including 68 trainees, to showcase the most recent discoveries and to highlight critical open questions in the field. De novo gene birth is now recognized as a process with profound effects on genome evolution and phenotypic innovation. The transformative results generated from ribosome profiling have further emphasized the key role of de novo gene birth in producing novel proteins and functional peptides. Experimental analyses of individual de novo genes are illuminating their biological roles, and early investigations on the structure of interactions of de novo proteins are contributing to reveal their interactions with other cellular components. Exciting progress in this field should be gained by further integrating comparative genomics, functional genomics, structural proteomics and systems biology across experimental models. Additional theoretical work will be necessary to elucidate the complex evolutionary dynamics leading to de novo genes from ancestrally noncoding genomic sequences. A core development will be the ability to compile extensive surveys of de novo genes across thousands of genomes, thus revealing their full biological impact along the tree of life.

## Data Availability

No new data were generated or analyzed in support of this manuscript.
